# Some New Generalized Difference Spaces of Nonabsolute Type Derived from the Spaces *ℓ*_*p*_ and *ℓ*_*∞*_

**DOI:** 10.1155/2013/349346

**Published:** 2013-11-14

**Authors:** Feyzi Başar, Ali Karaisa

**Affiliations:** ^1^Department of Mathematics, Faculty of Arts and Sciences, Fatih University, Hadımköy Campus, Büyükçekmece, 34500 İstanbul, Turkey; ^2^Department of Mathematics & Computer Science, Faculty of Science, Necmettin Erbakan University, Meram Campus, Meram, 42090 Konya, Turkey

## Abstract

We introduce the sequence space *ℓ*_*p*_^*λ*^(*B*) of none absolute type which is a *p*-normed space and *BK* space in the cases 0 < *p* < 1 and 1 ⩽ *p* ⩽ *∞*, respectively, and prove that *ℓ*_*p*_^*λ*^(*B*) and *ℓ*_*p*_ are linearly isomorphic for 0 < *p* ⩽ *∞*. Furthermore, we give some inclusion relations concerning the space *ℓ*_*p*_^*λ*^(*B*) and we construct the basis for the space *ℓ*_*p*_^*λ*^(*B*), where 1 ⩽ *p* < *∞*. Furthermore, we determine the alpha-, beta- and gamma-duals of the space *ℓ*_*p*_^*λ*^(*B*) for 1 ⩽ *p* ⩽ *∞*. Finally, we investigate some geometric properties concerning Banach-Saks type *p* and give Gurarii's modulus of convexity for the normed space *ℓ*_*p*_^*λ*^(*B*).

## 1. Introduction

From the summability theory perspective, the role played by the algebraical, geometrical, and topological properties of the new Banach spaces which are defined by the matrix domain of triangle matrices in sequence spaces is well-known.

By *w*, we denote the space of all real or complex valued sequences. Any vector subspace of *w* is called a sequence space.

A sequence space *μ* with a linear topology is called a *K*-space provided that each of the maps *p*_*i*_ : *μ* → *ℂ* defined by *p*_*i*_(*x*) = *x*_*i*_ is continuous for all *i* ∈ *ℕ*, where *ℂ* denotes the complex field and *ℕ* = {0,1, 2,…}. A *K*-space is called an *FK*-space provided *μ* is a complete linear metric space. An *FK*-space whose topology is normable is called a *BK*-space (see [1]) which contains *ϕ*, the set of all finitely nonzero sequences.

We write *ℓ*_*∞*_, *f*, *c*, and *c*_0_ for the spaces of all bounded, almost convergent, convergent, and null sequences, respectively, which are *BK*-spaces with the usual supnorm defined by

(1) $$\begin{array}{c} {||x||}_{\infty }=\underset{k\in \mathbb{N}}{\sup}\left|x_{k}\right|. \end{array}$$

Also, by *ℓ*_*p*_ and *ℓ*_1_, we denote the spaces all *p*-absolutely and absolutely convergent series, respectively, which are *BK*-spaces with the usual norm defined by

(2) $$\begin{array}{c} {||x||}_{p}={\left(\sum_{k}{\left|x_{k}\right|}^{p}\right)}^{1/p},\;\left(1⩽p<\infty \right). \end{array}$$

Here, and in what follows, the summation without limits runs from 0 to *∞*. Further, we write bs and cs for the spaces of all bounded and convergent series, respectively, which are *BK*-spaces with their natural norm [2].

Let *μ* and *γ* be two sequence spaces and *A* = (*a*_*nk*_) be an infinite matrix of real or complex numbers *a*_*nk*_, where *n*, *k* ∈ *ℕ*. Then, we say that *A* defines a matrix transformation from *μ* into *γ* and we denote it by writing *A* : *μ* → *γ*, if for every sequence *x* = (*x*_*k*_) ∈ *μ* the sequence *Ax* = {(*Ax*)_*n*_}, the *A*-transform of *x* is in *γ*, where

(3) $$\begin{array}{c} {\left(Ax\right)}_{n}=\sum_{k}a_{nk}x_{k}\;\forall n\in \mathbb{N}. \end{array}$$

The notation (*μ* : *γ*) denotes the class of all matrices *A* such that *A* : *μ* → *γ*. Thus, *A* ∈ (*μ* : *γ*) if and only if the series on the right hand side of (3) converges for each *n* ∈ *ℕ* and every *x* ∈ *μ*, and we have *Ax* = {(*Ax*)_*n*_}_*n*∈*ℕ*_ ∈ *γ* for all *x* ∈ *μ*. The matrix domain *μ*_*A*_ of an infinite matrix *A* in a sequence space *μ* is defined by

(4) $$\begin{array}{c} \mu_{A}=\left\{x=\left(x_{k}\right)\in \omega :Ax\in \mu \right\}. \end{array}$$

An infinite matrix *A* = (*a*_*nk*_) is said to be a triangle if *a*_*nn*_ ≠ 0 for all *n* ∈ *ℕ* and *a*_*nk*_ = 0 for *k* > *n*. The study of matrix domains of triangles has a special importance due to the various properties which they have. For example, if *A* is a triangle and *μ* is a *BK*-space, then *μ*_*A*_ is also a *BK*-space with the norm given by ||*x*||_*μ*_*A*__ = ||*Ax*||_*μ*_ for all *x* ∈ *μ*_*A*_.

Throughout the paper, we denote the collection of all finite subsets of *ℕ* by *ℱ*. Also, we write *e* ^(*k*)^ for the sequence whose only nonzero term is a 1 in the *k*th place for each *k* ∈ *ℕ*.

The approach constructing a new sequence space by means of the matrix domain of a particular triangle has recently been employed by several authors in many research papers. For example, they introduced the sequence spaces (*ℓ*_*∞*_)_*N*_*q*__ and *c*_*N*_*q*__ in [3], (*ℓ*_*p*_)_*C*_1__ = *X*_*p*_ and (*ℓ*_*∞*_)_*C*_1__ = *X*_*∞*_ in [4], ${\left(c_{0}\right)}_{C_{1}}={\tilde{c}}_{0}$ and ${\left(c\right)}_{C_{1}}=\tilde{c}$ in [5], (*ℓ*_*p*_)_*E*^*r*^_ = *e*_*p*_^*r*^ and (*ℓ*_*∞*_)_*E*^*r*^_ = *e*_*∞*_^*r*^ in [6], (*ℓ*_*p*_)_*A*^*r*^_ = *a*_*p*_^*r*^ and (*ℓ*_*∞*_)_*A*^*r*^_ = *a*_*∞*_^*r*^ in [7], (*ℓ*_*p*_)_Δ_ = *bv*_*p*_ and (*ℓ*_*∞*_)_Δ_ = *bv*_*∞*_ in [8], *μ*_*G*_ = *Z*(*u*, *v*; *μ*) in [9], (*c*_0_)_Λ_ = *c*_0_^*λ*^ and *c*_Λ_ = *c* ^*λ*^ in [10], and (*ℓ*_*p*_)_Δ^(*m*)^_ = *ℓ*_*p*_(Δ^(*m*)^) in [11], where *N*_*q*_, *C*_1_, *R* ^*t*^, *E* ^*r*^, *A* ^*r*^, Λ, and Δ^(*m*)^ denote Nörlund, arithmetic, Riesz, Euler means, *A* ^*r*^ matrix, lambda matrix, and generalized difference matrix, respectively, where 1 ⩽ *p* < *∞*.

Recently, there has been a lot of interest in investigating geometric properties of sequence spaces besides topological and some other usual properties. In the literature, there are many papers concerning the geometric properties of different sequence spaces. For example, in [12], Mursaleen et al. studied some geometric properties of a normed Euler sequence space. Recently, Şimşek and Karakaya [13] have investigated the geometric properties of the sequence space *ℓ*(*u*, *v*, *p*) equipped with Luxemburg norm. Later, Demiriz and Çakan [14] have studied some geometric properties of the sequence space *a*_*p*_^*r*^(Δ). For further information on geometric properties of sequence spaces the reader can refer to [15, 16].

The main purpose of the present paper is to introduce the difference sequence spaces *ℓ*_*p*_^*λ*^(*B*) of nonabsolute type and derive some related results. We also establish some inclusion relations, where 0 < *p* ⩽ *∞*. Furthermore, we determine the alpha-, beta- and gamma-duals of those spaces and construct their bases. We characterize some classes of infinite matrices concerning the spaces *ℓ*_*p*_^*λ*^(*B*) and *ℓ*_*∞*_^*λ*^(*B*) for 1 ⩽ *p* < *∞*. Finally, we investigate some geometric properties concerning Banach-Saks type *p* and give Gurarii's modulus of convexity for the normed space *ℓ*_*p*_^*λ*^(*B*).

## 2. The Sequence Spaces *ℓ*_*p*_^*λ*^(*B*) and *ℓ*_*∞*_^*λ*^(*B*) of Nonabsolute Type

This section is devoted to the examination of the basic topological properties of the sets *ℓ*_*p*_^*λ*^(*B*) and *ℓ*_*∞*_^*λ*^(*B*). Let throughout that (*λ*_*k*_) be strictly increasing sequence of positive reals tending to *∞*; that is

(5) $$\begin{array}{c} 0<\lambda_{1}<\lambda_{2}<\cdots ,\;\;\underset{k\to \infty }{\lim}\lambda_{k}=\infty . \end{array}$$

Let us define the lambda matrix Λ = (*λ*_*nk*_) by

(6) $$\begin{array}{c} \lambda_{nk}=\{\begin{array}{cc} \frac{\lambda_{k}-\lambda_{k-1}}{\lambda_{n}}, & 0⩽k⩽n,\\ 0, & k>n. \end{array} \end{array}$$

Recently, Mursaleen and Noman [17, 18] have studied the sequence spaces *ℓ*_*∞*_^*λ*^ and *ℓ*_*p*_^*λ*^ of nonabsolute type as follows:

(7) ℓpλ:={x=(xk)∈ω:∑n|∑k=0nλk−λk−1λnxk|p<∞};(0<p<∞),ℓ∞λ:={x=(xk)∈ω:sup⁡n∈ℕ|∑k=0nλk−λk−1λnxk|<∞}.

With the notation of (4), we can redefine the spaces *ℓ*_*∞*_^*λ*^ and *ℓ*_*p*_^*λ*^ by *ℓ*_*p*_^*λ*^ = (*ℓ*_*p*_)_Λ_ and *ℓ*_*∞*_^*λ*^ = (*ℓ*_*∞*_)_Λ_, where 0 < *p* < *∞*.

Let *r* and *s* be non-zero real numbers, and define the generalized difference matrix *B*(*r*, *s*) = {*b*_*nk*_(*r*, *s*)} by

(8) $$\begin{array}{c} b_{nk}\left(r,s\right)=\{\begin{array}{cc} r, & k=n,\\ s, & k=n-1,\\ 0, & \text{otherwise}, \end{array} \end{array}$$

for all *n*, *k* ∈ *ℕ*. The *B*(*r*, *s*)-transform of a sequence *x* = (*x*_*k*_) is {*B*(*r*, *s*)*x*}_*k*_ = *rx*_*k*_ + *sx*_*k*−1_ for all *k* ∈ *ℕ*. We note that the matrix *B*(*r*, *s*) can be reduced to the difference matrix Δ in the case *r* = 1 and *s* = −1. So, the results related to the domain of the matrix *B*(*r*, *s*) are more general and comprehensive than those of the matrix domain of Δ and include them.

Now, we introduce the new sequence spaces *ℓ*_*p*_^*λ*^(*B*) and *ℓ*_*∞*_^*λ*^(*B*) as follows:

(9) ℓpλ(B):={x=(xk)∈ω:∑n|∑k=0nλk−λk−1λn(rxk+sxk−1)|p<∞};(0<p<∞),ℓ∞λ(B):={x=(xk)∈ω:sup⁡n∈ℕ|∑k=0nλk−λk−1λn(rxk+sxk−1)|<∞}.

By the notation of (4), we can redefine the spaces *ℓ*_*∞*_^*λ*^(*B*) and *ℓ*_*p*_^*λ*^(*B*) as follows:

(10) $$\begin{array}{c} \ell_{p}^{\lambda }\left(B\right)={\left(\ell_{p}^{\lambda }\right)}_{B}\;\left(0<p<\infty \right),\;\;\ell_{\infty }^{\lambda }\left(B\right)={\left(\ell_{\infty }^{\lambda }\right)}_{B}, \end{array}$$

where *B* denotes the generalized difference matrix *B*(*r*, *s*) = {*b*_*nk*_(*r*, *s*)} defined by (8). Now, we may define the triangle matrix $\hat{\Lambda }=({\hat{\lambda }}_{nk})$ by

(11) $$\begin{array}{c} {\hat{\lambda }}_{nk}=\{\begin{array}{cc} \frac{r\left(\lambda_{k}-\lambda_{k-1}\right)+s\left(\lambda_{k+1}-\lambda_{k}\right)}{\lambda_{n}}, & k<n,\\ \frac{r\left(\lambda_{n}-\lambda_{n-1}\right)}{\lambda_{n}}, & k=n,\\ 0, & k>n. \end{array} \end{array}$$

Define the sequence *y* = (*y*_*k*_) as the $\hat{\Lambda }$-transform of a sequence *x* = (*x*_*k*_); that is,

(12) yk=(Λ^x)k=∑i=0k−1r(λi−λi−1)+s(λi+1−λi)λkxi +r(λk−λk−1)λkxk ∀k∈ℕ.

Now, we can redefine the spaces *ℓ*_*∞*_^*λ*^(*B*) and *ℓ*_*p*_^*λ*^(*B*) with the notation of (4) as

(13) $$\begin{array}{c} \ell_{p}^{\lambda }\left(B\right)={\left(\ell_{p}\right)}_{\hat{\Lambda }}\;\left(0<p<\infty \right),\;\;\ell_{\infty }^{\lambda }\left(B\right)={\left(\ell_{\infty }\right)}_{\hat{\Lambda }}. \end{array}$$

Also, we derive from the equality (12) that

(14) $$\begin{array}{c} y_{k}=\sum_{i=0}^{k}\frac{\left(\lambda_{i}-\lambda_{i-1}\right)\left(rx_{i}+sx_{i-1}\right)}{\lambda_{k}}\;\forall k\in \mathbb{N}. \end{array}$$

Then, since the sequence spaces *ℓ*_*p*_^*λ*^(*B*) and *ℓ*_*p*_ are linearly isomorphic; that is, *ℓ*_*p*_^*λ*^(*B*)≅*ℓ*_*p*_; it is trivial that the two-sided implication “*x* ∈ *ℓ*_*p*_^*λ*^(*B*) if and only if *y* ∈ *ℓ*_*p*_” holds, where 0 < *p* ⩽ *∞*.

We have the following result which is essential in the text.

**Theorem 1**

The following statements hold.

(a) If 0 < *p* < 1, then *ℓ*_*p*_^*λ*^(*B*) is a complete *p*-normed space with the *p*-norm ${||x||}_{\ell_{p}^{\lambda }(B)}={||\hat{\Lambda }(x)||}_{p}$; that is,
  (15) $$\begin{array}{c} {||x||}_{\ell_{p}^{\lambda }(B)}=\sum_{n}{\left|{\left(\hat{\Lambda }x\right)}_{n}\right|}^{p};\;\left(0<p<1\right). \end{array}$$
(b) If 1 ⩽ *p* ⩽ *∞*, then *ℓ*_*p*_^*λ*^(*B*) is *BK*-space with the norm ${||x||}_{\ell_{p}^{\lambda }(B)}={||\hat{\Lambda }(x)||}_{p}$; that is,
  (16) $$\begin{array}{c} {||x||}_{\ell_{p}^{\lambda }(B)}={\left[\sum_{n}{\left|{\left(\hat{\Lambda }x\right)}_{n}\right|}^{p}\right]}^{1/p};\;\left(1⩽p<\infty \right), \end{array}$$
  (17) $$\begin{array}{c} {||x||}_{\ell_{\infty }^{\lambda }(B)}=\underset{n\in \mathbb{N}}{\sup}\left|{\left(\hat{\Lambda }x\right)}_{n}\right|. \end{array}$$

**Proof**

(a) Let 0 < *p* < 1. It is immediate by the fact *ℓ*_*p*_^*λ*^(*B*)≅*ℓ*_*p*_ that *ℓ*_*p*_^*λ*^(*B*) is a complete *p*-normed linear space with the *p*-norm ${||x||}_{\ell_{p}^{\lambda }(B)}=\sum_{n}{\left|{\left(\hat{\Lambda }x\right)}_{n}\right|}^{p}$.

(b) Since the sets *ℓ*_*p*_ and *ℓ*_*∞*_ endowed with the norms ||·||_*p*_ and ||·||_*∞*_ are *BK*-spaces (see [2, Example 7.3.2 (b), (c)]) and the matrix $\hat{\Lambda }$ is triangle, Theorem 4.3.2 of Wilansky [19, page 61] gives the fact that the spaces *ℓ*_*p*_^*λ*^(*B*) and *ℓ*_*∞*_^*λ*^(*B*) are *BK*-spaces with the norms in (16) and (17), respectively.

One can easily check that the absolute property does not hold on the space *ℓ*_*p*_^*λ*^(*B*); that is, ||*x*||_*ℓ*_*∞*_^*λ*^(*B*)_ ≠ |||*x*|||_*ℓ*_*∞*_^*λ*^(*B*)_ for at least one sequence in the space *ℓ*_*p*_^*λ*^(*B*), and this tells us that *ℓ*_*p*_^*λ*^(*B*) is nonabsolute type, where |*x* | = (|*x*_*k*_|) and 0 < *p* ⩽ *∞*.

Now, one may expect the similar result for the space *ℓ*_*p*_^*λ*^(*B*) as was observed for the space *ℓ*_*p*_ and ask the natural question: Is not the space *ℓ*_*p*_^*λ*^(*B*) a Hilbert space with *p* ≠ 2? The answer is positive and is given by the following theorem.

**Theorem 2**

Except the case *p* = 2, the space *ℓ*_*p*_^*λ*^(*B*) is not an inner product space and hence it is not a Hilbert space, where 1 ⩽ *p* < *∞*.

**Proof**

We have to prove that the space *ℓ*_2_^*λ*^(*B*) is the only Hilbert space among the *ℓ*_*p*_^*λ*^(*B*) spaces for 1 ⩽ *p* < *∞*. Since the space *ℓ*_*p*_^*λ*^(*B*) is a *BK*-space with the norm ${||x||}_{\ell_{2}^{\lambda }(B)}={||\hat{\Lambda }x||}_{2}$ by Part (b) of Theorem 1 and its norm can be obtained from an inner product; that is,

(18) $$\begin{array}{c} {||x||}_{\ell_{2}^{\lambda }(B)}={\langle x,x\rangle }^{1/2}={\langle \hat{\Lambda }x,\hat{\Lambda }x\rangle }^{1/2} \end{array}$$

holds for every *x* ∈ *ℓ*_2_^*λ*^(*B*), the space *ℓ*_2_^*λ*^(*B*) is a Hilbert space. Let us define the sequences *z* = (*z*_*k*_) and *t* = (*t*_*k*_) by

(19) $$\begin{array}{c} z_{k}:=\{\begin{array}{cc} \frac{1}{r},k=0, & \\ \frac{r-s}{r^{2}},k=1, & \\ {\left(\frac{-s}{r}\right)}^{k-2}\frac{1}{r}\left[\left(\frac{-s}{r}\right)+{\left(\frac{-s}{r}\right)}^{2}-\left(\frac{\lambda_{1}}{\lambda_{2}-\lambda_{1}}\right)\right], & \\ k⩾2, & \end{array}\\ t_{k}:=\{\begin{array}{cc} \frac{1}{r},k=0, & \\ -\frac{1}{r}\left(\frac{\lambda_{1}+\lambda_{0}}{\lambda_{1}-\lambda_{0}}+\frac{s}{r}\right),k=1, & \\ {\left(\frac{-s}{r}\right)}^{k-2}\frac{1}{r}\left[\frac{\lambda_{1}}{\lambda_{2}-\lambda_{1}}+{\left(\frac{s}{r}\right)}^{2}+\frac{s}{r}\left(\frac{\lambda_{1}+\lambda_{0}}{\lambda_{1}-\lambda_{0}}\right)\right], & \\ k⩾2. & \end{array} \end{array}$$

Then, we have

(20) $$\begin{array}{c} \hat{\Lambda }z=\left(1,1,0,0,\ldots \right),\;\;\hat{\Lambda }t=\left(1,-1,0,0,\ldots \right). \end{array}$$

Thus, it can easily be seen that

(21) ||z+t||ℓpλ(B)2+||z−t||ℓpλ(B)2=8≠4(22/p)=2(||z||ℓpλ(B)2+||t||ℓpλ(B)2); (p≠2),

that is, the norm of the space *ℓ*_*p*_^*λ*^(*B*) with *p* ≠ 2 does not satisfy the parallelogram equality which means that this norm cannot be obtained from an inner product. Hence, the space *ℓ*_*p*_^*λ*^(*B*) with *p* ≠ 2 is a Banach space which is not a Hilbert space. This completes the proof.

## 3. The Inclusion Relations

In this section, we give some inclusion relations between the spaces *ℓ*_*p*_ and *ℓ*_*∞*_ and the spaces *ℓ*_*p*_^*λ*^(*B*) and *ℓ*_*∞*_^*λ*^(*B*), where 0 < *p* < *∞*. We essentially prove that *ℓ*_*∞*_ ⊂ *ℓ*_*∞*_^*λ*^(*B*) holds and characterize the case in which the inclusion *ℓ*_*p*_ ⊂ *ℓ*_*p*_^*λ*^(*B*) holds for 1 ⩽ *p* < *∞*.

**Theorem 3**

Let 0 < *p* < *s* < *∞*. Then, the inclusions *ℓ*_*p*_^*λ*^(*B*) ⊂ *ℓ*_*s*_^*λ*^(*B*) strictly holds.

**Proof**

Let 0 < *p* < *s* < *∞* and *x* = (*x*_*k*_) ∈ *ℓ*_*p*_^*λ*^(*B*). This implies that $\hat{\Lambda }x\in \ell_{p}$. Since *ℓ*_*p*_ ⊂ *ℓ*_*s*_, $\hat{\Lambda }x\in \ell_{s}$. So, we have *ℓ*_*p*_^*λ*^(*B*) ⊂ *ℓ*_*s*_^*λ*^(*B*).

Let us consider the sequence *z* = (*z*_*k*_) with the aid of the sequence *x* = (*x*_*k*_) = {(*k* + 1)^−(*s*+1−*p*)^}_*k*=0_^*∞*^ ∈ *ℓ*_*s*_∖*ℓ*_*p*_ defined by

(22) zk:=1r∑j=0k(−sr)k−j∑i=j−1j(−1)j−iλi(λj−λj−1)(i+1)−(s+1−p),∀k∈ℕ.

Then, since $\hat{\Lambda }z=x\in \ell_{s}\setminus \ell_{p},\;\;z\in \ell_{s}^{\lambda }(B)\setminus \ell_{p}^{\lambda }(B)$ which shows that the inclusion *ℓ*_*p*_^*λ*^(*B*) ⊂ *ℓ*_*s*_^*λ*^(*B*) is strict.

**Theorem 4**

The inclusions *ℓ*_*p*_^*λ*^(*B*) ⊂ *c*_0_^*λ*^(*B*) ⊂ *c* ^*λ*^(*B*) ⊂ *ℓ*_*∞*_^*λ*^(*B*) strictly hold.

**Proof**

The inclusion *c*_0_^*λ*^(*B*) ⊂ *c* ^*λ*^(*B*) strictly holds by Theorem 3.1 of [20]. So, it is enough to show that the inclusions *ℓ*_*p*_^*λ*^(*B*) ⊂ *c*_0_^*λ*^(*B*) and *c* ^*λ*^(*B*) ⊂ *ℓ*_*∞*_^*λ*^(*B*) are strict, where 0 < *p* < *∞*. Assume that *x* = (*x*_*k*_) ∈ *ℓ*_*p*_^*λ*^(*B*). This means that $\hat{\Lambda }x\in \ell_{p}$. Since *ℓ*_*p*_ ⊂ *c*_0_, $\hat{\Lambda }x\in c_{0}$ which implies that *x* ∈ *c*_0_^*λ*^(*B*). Hence, the inclusion *ℓ*_*p*_^*λ*^(*B*) ⊂ *c*_0_^*λ*^(*B*) holds for 0 < *p* < *∞*. Now, we show that this inclusion is strict. Let 0 < *p* < *∞* and define the sequence *x* = (*x*_*k*_) as follows:

(23) xk:=1r∑j=0k(−sr)k−j∑i=j−1j(−1)j−iλi(λj−λj−1)(i+1)1/pfor  each  k∈ℕ.

Then, we have for every *n* ∈ *ℕ* that

(24) (Λ^x)n:=1λn∑k=0n(λk−λk−1)(rxk+sxk−1)=1(n+1)1/p,

which shows that $\hat{\Lambda }x\notin \ell_{p}$ but $\hat{\Lambda }x\in c_{0}$. Thus, the sequence *x* is in *c*_0_^*λ*^(*B*) but not in *ℓ*_*p*_^*λ*^(*B*). Hence, *ℓ*_*p*_^*λ*^(*B*) ⊂ *c*_0_^*λ*^(*B*) is strict.

Since *c* ⊂ *ℓ*_*∞*_ holds, we have the inclusion *c* ^*λ*^(*B*) ⊂ *ℓ*_*∞*_^*λ*^(*B*). Let us define the sequence *y* = (*y*_*k*_) by

(25) yk:=1r∑j=0k(−sr)k−j∑i=j−1j(−1)j−iλiλj−λj−1(−1)ifor  each  k∈ℕ.

Then, one can easily see for every *n* ∈ *ℕ* that

(26) $$\begin{array}{c} {\left(\hat{\Lambda }y\right)}_{n}:=\frac{1}{\lambda_{n}}\sum_{k=0}^{n}\left(\lambda_{k}-\lambda_{k-1}\right)\left(rx_{k}+sx_{k-1}\right)={\left(-1\right)}^{n}, \end{array}$$

which shows that $\hat{\Lambda }y\in \ell_{\infty }\setminus c$. Thus, *y* is in *ℓ*_*∞*_^*λ*^(*B*) but not in *c* ^*λ*^(*B*); that is, *y* ∈ *ℓ*_*∞*_^*λ*^(*B*)∖*c* ^*λ*^(*B*). That is to say that the inclusion *c* ^*λ*^(*B*) ⊂ *ℓ*_*∞*_^*λ*^(*B*) is strict. This completes the proof.

**Theorem 5**

The inclusion *ℓ*_*∞*_ ⊂ *ℓ*_*∞*_^*λ*^(*B*) strictly holds.

**Proof**

Let *x* = (*x*_*k*_) ∈ *ℓ*_*∞*_. Then, we have

(27) ||x||ℓ∞λ(B)=sup⁡n∈ℕ|(Λ^x)n|=sup⁡n∈ℕ|1λn∑k=0n(λk−λk−1)(rxk+sxk−1)|⩽sup⁡n∈ℕ1λn∑k=0n(λk−λk−1)|sxk−1+rxk|⩽sup⁡n∈ℕ1λn∑k=0n(λk−λk−1)(|s||xk−1|+|r||xk|)    ⩽(|r|+|s|)||x||∞sup⁡n∈ℕ1λn∑k=0n(λk−λk−1)=(|r|+|s|)||x||∞,

which means that *x* = (*x*_*k*_) ∈ *ℓ*_*∞*_^*λ*^(*B*). So, the inclusion *ℓ*_*∞*_ ⊂ *ℓ*_*∞*_^*λ*^(*B*) holds. Furthermore, consider the sequence *v* = (*v*_*k*_) defined by

(28) $$\begin{array}{c} v_{k}:=\frac{1}{r}\sum_{i=0}^{k}{\left(\frac{-s}{r}\right)}^{k-i}\;\forall k\in \mathbb{N}\;\;\text{with}\;\left|-\frac{s}{r}\right|>1. \end{array}$$

Clearly, *v* ∉ *ℓ*_*∞*_. Then, we obtain by (12) that

(29) $$\begin{array}{c} {\left(\hat{\Lambda }v\right)}_{n}:=\frac{1}{\lambda_{n}}\sum_{k=0}^{n}\left(\lambda_{k}-\lambda_{k-1}\right)=1,\;\forall n\in \mathbb{N}, \end{array}$$

which shows that $\hat{\Lambda }v=e\in \ell_{\infty }$. This implies that *v* ∈ *ℓ*_*∞*_^*λ*^(*B*)∖*ℓ*_*∞*_. Hence, the inclusion *ℓ*_*∞*_ ⊂ *ℓ*_*∞*_^*λ*^(*B*) is strict and this completes the proof.

**Theorem 6**

If the inclusion *ℓ*_*p*_ ⊂ *ℓ*_*p*_^*λ*^(*B*) holds, then (1/*λ*_*n*_) ∈ *ℓ*_*p*_, where 0 < *p* < *∞*.

**Proof**

Assume that the inclusion *ℓ*_*p*_ ⊂ *ℓ*_*p*_^*λ*^(*B*) holds and consider sequence *e* ^(0)^ = (1,0, 0,…) ∈ *ℓ*_*p*_. So, by this hypothesis, *e* ^(0)^ = (1,0, 0,…) ∈ *ℓ*_*p*_^*λ*^(*B*). Hence, $\hat{\Lambda }e^{\left(0\right)}\in \ell_{p}$. Therefore,

(30) $$\begin{array}{c} \frac{1}{\lambda_{n}}\sum_{k=0}^{n}\left(\lambda_{k}-\lambda_{k-1}\right)\left(rx_{k}+sx_{k-1}\right)=\frac{1}{\lambda_{n}}\lambda_{0}r, \end{array}$$

and we obtain that

(31) $$\begin{array}{c} \sum_{n}{\left|{\left(\hat{\Lambda }e^{\left(0\right)}\right)}_{n}\right|}^{p}={\left|\lambda_{0}r\right|}^{p}\sum_{n}{\left(\frac{1}{\lambda_{n}}\right)}^{p}<\infty , \end{array}$$

which shows that (1/*λ*_*n*_) ∈ *ℓ*_*p*_. This completes the proof.

**Lemma 7 (see [17, Lemma 4.11, page, 43])**

If (1/*λ*_*n*_) ∈ *ℓ*_1_, then

(32) $$\begin{array}{c} M=\underset{n\in \mathbb{N}}{\sup}\sum_{n=k}^{\infty }\frac{\lambda_{k}-\lambda_{k-1}}{\lambda_{n}}<\infty . \end{array}$$

**Theorem 8**

If (1/*λ*_*n*_) ∈ *ℓ*_1_, the inclusion *ℓ*_*p*_ ⊂ *ℓ*_*p*_^*λ*^(*B*) strictly holds for 1 ⩽ *p* < *∞*.

**Proof**

Let *x* = (*x*_*k*_) ∈ *ℓ*_*p*_ for 1 < *p* < *∞*. Then, by applying Hölder's inequality, we derive from (12) that

(33) |(Λ^x)n|=|1λn∑k=0n(λk−λk−1)(rxk+sxk−1)|⩽∑k=0nλk−λk−1λn|sxk−1+rxk|=∑k=0n(λk−λk−1λn)1/p ×(λk−λk−1λn)p−1/p|sxk−1+rxk|⩽{∑k=0n[(λk−λk−1λn)1/p|sxk−1+rxk|]p}1/p×{∑k=0n[(λk−λk−1λn)p−1/p]q}1/q=[∑k=0n(λk−λk−1λn)|sxk−1+rxk|p]1/p×[∑k=0n(λk−λk−1λn)]p−1/p,

which gives that

(34) |(Λ^x)n|p ⩽∑k=0n(λk−λk−1λn)|sxk−1+rxk|p[∑k=0n(λk−λk−1λn)]p−1.

By (34) and Lemma 7, we have

(35) ∑n|(Λ^x)n|p⩽∑n1λn∑k=0n(λk−λk−1)|sxk−1+rxk|p=∑k|sxk−1+rxk|p∑n=k∞λk−λk−1λn⩽M∑k|sxk−1+rxk|p.

Therefore, combining the inequality (35) with Minkowski's inequality, we derive that

(36) ||x||ℓpλ(B)=(∑n|(Λ^x)n|p)1/p⩽M1/p|r|(∑k|xk|p)1/p+M1/p|s|(∑k|xk−1|p)1/p⩽M1/p(|r|+|s|)||x||p<∞.

This shows that *x* ∈ *ℓ*_*p*_^*λ*^(*B*). So, the inclusion *ℓ*_*p*_ ⊂ *ℓ*_*p*_^*λ*^(*B*) holds. Now, let us consider the sequence *v* = (*v*_*k*_) defined by

(37) $$\begin{array}{c} v_{k}:=\{\begin{array}{cc} \frac{1}{r}, & k=0,\\ -\frac{1}{r}\left(\frac{\lambda_{0}}{\lambda_{1}-\lambda_{0}}+\frac{s}{r}\right){\left(-\frac{s}{r}\right)}^{k-1}, & k⩾1, \end{array} \end{array}$$

with |−*s*| > |*r*|. Then, since $\hat{\Lambda }v=e^{\left(0\right)}\in \ell_{p}$, one can immediately observe that *v* is in *ℓ*_*p*_^*λ*^(*B*) but not in *ℓ*_*p*_. That is, *v* ∈ *ℓ*_*p*_^*λ*^(*B*)∖*ℓ*_*p*_. Thus, we have showed that the inclusion *ℓ*_*p*_ ⊂ *ℓ*_*p*_^*λ*^(*B*) is strict. Similarly, the inclusion *ℓ*_*p*_ ⊂ *ℓ*_*p*_^*λ*^(*B*) also strictly holds in the case *p* = 1, so we omit the details. This completes the proof.

**Theorem 9**

The sequence spaces *ℓ*_*∞*_ and *ℓ*_*p*_^*λ*^(*B*) do not include each other.

**Proof**

It is clear by Theorem 8 that the sequence spaces *ℓ*_*∞*_ and *ℓ*_*p*_^*λ*^(*B*) are not disjointed. Let us consider the sequence *v* = (*v*_*k*_) defined by (37). Then, *v* is in *ℓ*_*p*_^*λ*^(*B*) but not in *ℓ*_*∞*_. Now, let us define the sequence *x* = (*x*_*k*_): = {(1/*r*)∑_*i*=0_^*k*^(−*s*  /*r*)^*k*−*i*^}_*k*∈*ℕ*_ with |−*s*/*r*| < 1. Then, since $\hat{\Lambda }x=e\notin \ell_{p}$, *x* is in *ℓ*_*∞*_ but not in *ℓ*_*p*_^*λ*^(*B*). This completes the proof.

## 4. The Basis for the Space *ℓ*_*p*_^*λ*^(*B*)

In this section, we begin with defining the concept of the Schauder basis for a normed sequence space and then give the basis of the sequence space *ℓ*_*p*_^*λ*^(*B*), where 1 ⩽ *p* < *∞*. Now, we define the Schauder basis of a normed space. If a normed sequence space *μ* contains a sequence (*b*_*n*_) with the property that for every *x* ∈ *μ* there is a unique sequence of scalars (*α*_*n*_) such that

(38) $$\begin{array}{c} \underset{n\to \infty }{\lim}||x-\left(\alpha_{0}b_{0}+\alpha_{1}b_{1}+\cdots +\alpha_{n}b_{n}\right)||=0, \end{array}$$

then (*b*_*n*_) is called a *Schauder basis* (or briefly *basis*) for *μ*. The series ∑_*k*_ *α*_*k*_ *b*_*k*_ which has the sum *x* is called the expansion of *x* with respect to (*b*_*n*_), and written as *x* = ∑_*k*_ *α*_*k*_ *b*_*k*_.

**Theorem 10**

The following statements hold.

(i) The space *ℓ*_*∞*_^*λ*^(*B*) has no Schauder basis.
(ii) Define the sequence *b* ^(*k*)^ = {*b*_*n*_^(*k*)^}_*n*∈*ℕ*_ of elements of the space *ℓ*_*p*_^*λ*^(*B*) by
  (39) $$\begin{array}{c} b_{n}^{\left(k\right)}=\{\begin{array}{cc} {\left(\frac{-s}{r}\right)}^{n-k}\left[\frac{\lambda_{k}}{r\left(\lambda_{k}-\lambda_{k-1}\right)}+\frac{\lambda_{k}}{s\left(\lambda_{k+1}-\lambda_{k}\right)}\right], & k<n,\\ \frac{\lambda_{n}}{r\left(\lambda_{n}-\lambda_{n-1}\right)}, & k=n,\\ 0, & k>n, \end{array} \end{array}$$

for all *n*, *k* ∈ *ℕ*. Then, the sequence {*b*_*n*_^(*k*)^} is a basis for the space *ℓ*_*p*_^*λ*^(*B*) and every *x* ∈ *ℓ*_*p*_^*λ*^(*B*) has a unique representation of the form

(40) $$\begin{array}{c} x=\sum_{k}{\left(\hat{\Lambda }x\right)}_{k}b^{\left(k\right)}. \end{array}$$

**Proof**

(i) It is known that the matrix domain *μ*_*A*_ of a normed sequence space *μ* has a basis if and only if *μ* has a basis whenever *A* = (*a*_*nk*_) is a triangle [21, Remark 2.4]. Since the space *ℓ*_*∞*_ has no Schauder basis, *ℓ*_*∞*_^*λ*^(*B*) has no Schauder basis.

(ii) Let 1 ⩽ *p* < *∞*. It is clear that *b* ^(*k*)^ = {*b*_*n*_^(*k*)^} ⊂ *ℓ*_*p*_^*λ*^(*B*), since $\hat{\Lambda }b^{\left(k\right)}=e^{\left(k\right)}\in \ell_{p}$ for all *k* ∈ *ℕ*. Furthermore, let *x* ∈ *ℓ*_*p*_^*λ*^(*B*) be given. For every nonnegative integer *m*, we put

(41) $$\begin{array}{c} x^{\left[m\right]}=\sum_{k=0}^{m}{\left(\hat{\Lambda }x\right)}_{k}b^{\left(k\right)}. \end{array}$$

Then, by applying $\hat{\Lambda }$ to (41), we get that

(42) $$\begin{array}{c} \hat{\Lambda }x^{\left[m\right]}=\sum_{k=0}^{m}{\left(\hat{\Lambda }x\right)}_{k}\hat{\Lambda }b^{\left(k\right)}=\sum_{k=0}^{m}{\left(\hat{\Lambda }x\right)}_{k}e^{\left(k\right)}, \end{array}$$

and therefore, we have

(43) $$\begin{array}{c} {\left\{\hat{\Lambda }\left(x-x^{\left[m\right]}\right)\right\}}_{n}=\{\begin{array}{cc} 0, & 0⩽n⩽m,\\ {\left(\hat{\Lambda }x\right)}_{n}, & n>m, \end{array} \end{array}$$

for all *n*, *m* ∈ *ℕ*. Now, for any given *ϵ* > 0, there is a nonnegative integer *m*_0_ such that

(44) $$\begin{array}{c} \sum_{n=m_{0}+1}^{\infty }{\left|{\left(\hat{\Lambda }x\right)}_{n}\right|}^{p}<{\left(\frac{\epsilon }{2}\right)}^{p}. \end{array}$$

Thus, we have for every *m*⩾*m*_0_ that

(45) ||x−x[m]||ℓpλ(B) =(∑n=m+1∞|(Λ^x)n|p)1/p⩽(∑n=m0+1∞|(Λ^x)n|p)1/p⩽ϵ2<ϵ,

for all *m*⩾*m*_0_ which proves that *x* ∈ *ℓ*_*p*_^*λ*^(*B*) is represented as in (40).

Let us show that the uniqueness of representation for *x* ∈ *ℓ*_*p*_^*λ*^(*B*) is given by (40). Suppose, on the contrary, that there exists a representation *x* = ∑_*k*_ *μ*_*k*_(*x*)*b* ^(*k*)^. Since the transformation *T* defined from *ℓ*_*p*_^*λ*^(*B*) to *ℓ*_*p*_ by $Tx=\hat{\Lambda }x=y$ is continuous, we have

(46) $$\begin{array}{c} {\left(\hat{\Lambda }x\right)}_{k}=\sum_{n}\mu_{n}{\left\{\hat{\Lambda }b^{\left(k\right)}\right\}}_{n}=\sum_{n}\mu_{n}\left(x\right)\delta_{nk}=\alpha_{k}\left(x\right);\;k\in \mathbb{N}, \end{array}$$

which contradicts the assumption that ${\left(\hat{\Lambda }x\right)}_{k}\neq \alpha_{k}(x)$ for all *k* ∈ *ℕ*. That is to say that the representation (40) of *x* ∈ *ℓ*_*p*_^*λ*^(*B*) is unique.

## 5. The Alpha-, Beta- and Gamma-Duals of the Space *ℓ*_*p*_^*λ*^(*B*) and *ℓ*_*∞*_^*λ*^(*B*)

In this section, we give some theorems determining the alpha-, beta- and gamma-duals of the spaces *ℓ*_*p*_^*λ*^(*B*) and *ℓ*_*∞*_^*λ*^(*B*). We start with the definition of the alpha-, beta- and gamma-duals of a sequence space.

If *x* and *y* are sequences and *X* and *Y* are subsets of *ω*, then we write *x* · *y* = (*x*_*k*_ *y*_*k*_)_*k*=0_^*∞*^, *x* ^−1^∗*Y* = {*a* ∈ *ω* : *a* · *x* ∈ *Y*}, and

(47) $$\begin{array}{c} x\cdot y={\left(x_{k}y_{k}\right)}_{k=0}^{\infty },\;\;x^{-1}*Y=\left\{a\in \omega :a\cdot x\in Y\right\},\\ M\left(X,Y\right)=\underset{x\in X}{⋂}x^{-1}*Y=\left\{a:a\cdot x\in Y\;\;\forall x\in X\right\}, \end{array}$$

for the multiplier space of *X* and *Y*. One can easily observe for a sequence space *Z* with *Y* ⊂ *Z* and *Z* ⊂ *X* that the inclusions *M*(*X*, *Y*) ⊂ *M*(*X*, *Z*) and *M*(*X*, *Y*) ⊂ *M*(*Z*, *Y*) hold. The alpha-, beta- and gamma-duals of a sequence space, which are, respectively, denoted by *X* ^*α*^, *X* ^*β*^, and *X* ^*γ*^ are defined by

(48) $$\begin{array}{c} X^{\alpha }=M\left(X,\ell_{1}\right),\;{\;X}^{\beta }=M\left(X,\text{cs}\right),\;\;X^{\gamma }=M\left(X,\text{bs}\right). \end{array}$$

It is obvious that *X* ^*α*^ ⊂ *X* ^*β*^ ⊂ *X* ^*γ*^. Also, it can be easily seen that the inclusions *X* ^*α*^ ⊂ *Y* ^*α*^, *X* ^*β*^ ⊂ *Y* ^*β*^, and *X* ^*γ*^ ⊂ *Y* ^*γ*^ hold, whenever *Y* ⊂ *X*. Now, we may begin with quoting the following lemmas [22] which are needed in proving Theorems 14–16.

**Lemma 11**

*A* = (*a*_*nk*_)∈(*ℓ*_*p*_ : *ℓ*_1_) if and only if

(i) For 1 < *p* ⩽ *∞*
  (49) $$\begin{array}{c} \underset{F\in ℱ}{\sup}\sum_{k}{\left|\sum_{n\in F}a_{nk}\right|}^{q}<\infty . \end{array}$$
(ii) For *p* = 1
  (50) $$\begin{array}{c} \underset{k\in \mathbb{N}}{\sup}\sum_{n}\left|a_{nk}\right|<\infty . \end{array}$$

**Lemma 12**

Let *A* = (*a*_*nk*_) be an infinite matrix. Then, the following statements hold.

(i) Let 1 < *p* < *∞*. Then, *A* ∈ (*ℓ*_*p*_ : *c*) if and only if
  (51) $$\begin{array}{c} \underset{n\to \infty }{\lim}a_{nk}\;exists\;\;for\;\;each\;\;fixed\;\;k\in \mathbb{N}, \end{array}$$
  (52) $$\begin{array}{c} \underset{n\in \mathbb{N}}{\sup}\sum_{k}{\left|a_{nk}\right|}^{q}<\infty . \end{array}$$
(ii) *A* ∈ (*ℓ*_1_ : *c*) if and only if (51) holds and
  (53) $$\begin{array}{c} \underset{n,k\in \mathbb{N}}{\sup}\left|a_{nk}\right|<\infty . \end{array}$$
(iii) *A* ∈ (*ℓ*_*∞*_ : *c*) if and only if (51) holds and
  (54) $$\begin{array}{c} \underset{n\in \mathbb{N}}{\sup}\sum_{k}\left|a_{nk}\right|<\infty ,\\ \underset{n\to \infty }{\lim}\sum_{k}\left|a_{nk}-\underset{n\to \infty }{\lim}a_{nk}\right|=0. \end{array}$$

**Lemma 13**

Let *A* = (*a*_*nk*_) be an infinite matrix. Then, the following statements hold.

- Let 1 < *p* ⩽ *∞*. Then, *A* ∈ (*ℓ*_*p*_ : *ℓ*_*∞*_) if and only if (52) holds
- *A* ∈ (*ℓ*_1_ : *ℓ*_*∞*_) if and only if (53) holds.

**Theorem 14**

Define the sets *t*_*q*_^*λ*^ and *t*_*∞*_^*λ*^ by

(55) $$\begin{array}{c} t_{q}^{\lambda }=\left\{a=\left(a_{k}\right)\in \omega :\underset{F\in ℱ}{\sup}\sum_{k}{\left|\sum_{n\in F}t_{nk}^{\lambda }\right|}^{q}<\infty \right\},\\ t_{\infty }^{\lambda }=\left\{a=\left(a_{k}\right)\in \omega :\underset{k\in \mathbb{N}}{\sup}\sum_{n}\left|t_{nk}^{\lambda }\right|<\infty \right\}. \end{array}$$

Then, [*ℓ*_1_^*λ*^(*B*)]^*α*^ = *t*_*∞*_^*λ*^ and [*ℓ*_*p*_^*λ*^(*B*)]^*α*^ = *t*_*q*_^*λ*^ for 1 < *p* ⩽ *∞*, where the matrix *T* = (*t*_*nk*_^*λ*^) is defined via the sequence *a* = (*a*_*n*_) ∈ *w* by

(56) $$\begin{array}{c} t_{nk}^{\lambda }=\{\begin{array}{cc} {\left(\frac{-s}{r}\right)}^{n-k}\left[\frac{\lambda_{k}}{r\left(\lambda_{k}-\lambda_{k-1}\right)}+\frac{\lambda_{k}}{s\left(\lambda_{k+1}-\lambda_{k}\right)}\right]a_{n}, & \\ 0⩽k⩽n-1, & \\ \frac{\lambda_{n}}{r\left(\lambda_{n}-\lambda_{n-1}\right)}a_{n},k=n, & \\ 0,k>n, & \end{array} \end{array}$$

for all *n*, *k* ∈ *ℕ*.

**Proof**

Let *a* = (*a*_*n*_) ∈ *w* and 1 < *p* < *∞*. Then, by using (12), we immediately derive for every *n* ∈ *ℕ* that

(57) $$\begin{array}{c} a_{n}x_{n}=\frac{1}{r}\sum_{k=0}^{n}{\left(\frac{-s}{r}\right)}^{n-k}\sum_{i=k-1}^{k}{\left(-1\right)}^{k-i}\frac{\lambda_{i}}{\lambda_{k}-\lambda_{k-1}}y_{i}a_{n}={\left(Ty\right)}_{n}. \end{array}$$

Thus, we observe by (57) that *ax* = (*a*_*n*_ *x*_*n*_) ∈ *ℓ*_1_ whenever *x* = (*x*_*k*_) ∈ *ℓ*_*p*_^*λ*^(*B*) if and only if *Ty* ∈ *ℓ*_1_ whenever *y* = (*y*_*k*_) ∈ *ℓ*_*p*_. This means that *a* = (*a*_*k*_)∈[*ℓ*_*p*_^*λ*^(*B*)]^*α*^ if and only if *T* ∈ (*ℓ*_*p*_ : *ℓ*_1_). Therefore, we get by Lemma 11 with *T* instead of *A* that *a* = (*a*_*k*_)∈[*ℓ*_*p*_^*λ*^(*B*)]^*α*^ if and only if

(58) $$\begin{array}{c} \underset{F\in ℱ}{\sup}\sum_{k}{\left|\sum_{n\in F}t_{nk}^{\lambda }\right|}^{q}<\infty , \end{array}$$

which leads us to the consequence that [*ℓ*_*p*_^*λ*^(*B*)]^*α*^ = *t*_*q*_^*λ*^, for 1 < *p* ⩽ *∞*. Similarly, we get from (57) that *a* = (*a*_*k*_)∈[*ℓ*_1_^*λ*^(*B*)]^*α*^ if and only if *T* ∈ (*ℓ*_1_ : *ℓ*_1_) which is equivalent to (50) of Lemma 11 that

(59) $$\begin{array}{c} \underset{k\in \mathbb{N}}{\sup}\sum_{n}\left|t_{nk}^{\lambda }\right|<\infty . \end{array}$$

**Theorem 15**

Define the sets *d*_1_^*λ*^, *d*_2_^*λ*^, *d*_3_^*λ*^, *d*_4_^*λ*^, and *e*_*q*_^*λ*^ as follows:

(60) d1λ={a=(ak)∈ω:  ∑j=k+1∞(−sr)k−jaj  exists  for  each  k∈ℕ},eqλ={a=(ak)∈ω:sup⁡n∈ℕ∑k=0n−1|a~k(n)|q<∞},d2λ={a=(ak)∈ω:sup⁡k,n∈ℕ|a~k(n)|<∞},d3λ={a=(ak)∈ω:lim⁡n→∞∑k|a~k(n)|=∑k|a~k|},d4λ={a=(ak)∈ω:sup⁡k∈ℕ|λkr(λk−λk−1)ak|<∞},

where

(61) a~k(n)λk{akr(λk−λk−1)+[1r(λk−λk−1)+1s(λk+1−λk)]×∑j=k+1n(−sr)k−jaj} for  k<n,a~k=lim⁡n→∞a~k(n).

Then,

(62) $$\begin{array}{c} {\left[\ell_{p}^{\lambda }\left(B\right)\right]}^{\beta }=\{\begin{array}{cc} e_{1}^{\lambda }\cap d_{4}^{\lambda }\cap d_{3}^{\lambda }, & p=\;\infty ,\\ e_{q}^{\lambda }\cap d_{1}^{\lambda }\cap d_{4}^{\lambda }, & 1\;<p<\;\infty ,\\ d_{1}^{\lambda }\cap d_{2}^{\lambda }\cap d_{4}^{\lambda }, & p=\;1. \end{array} \end{array}$$

**Proof**

Let us consider the equality

(63) ∑k=0nakxk =∑k=0n{1r∑j=0k(−sr)k−j[∑i=j−1j(−1)j−iλiλj−λj−1yi]}ak =∑k=0n−1λk[(1r(λk−λk−1)+1s(λk+1−λk))  ×∑j=k+1n(−sr)k−jaj]yk+anr(λn−λn−1)yn =∑k=0n−1a~k(n)yk+anr(λn−λn−1)yn =(Dy)n ∀n∈ℕ,

where the matrix *D* = (*d*_*nk*_^*λ*^) is defined for all *n*, *k* ∈ *ℕ* by

(64) $$\begin{array}{c} d_{nk}^{\lambda }=\{\begin{array}{cc} {\tilde{a}}_{k}\left(n\right), & 0⩽k⩽n-1,\\ \frac{\lambda_{n}}{r\left(\lambda_{n}-\lambda_{n-1}\right)}a_{n}, & k=n,\\ 0, & k>n. \end{array} \end{array}$$

Then, we deduce from (63) with Lemma 12 that *ax* = (*a*_*n*_ *x*_*n*_) ∈ *cs* whenever *x* = (*x*_*k*_) ∈ *ℓ*_*p*_^*λ*^(*B*) if and only if *Dy* ∈ *c* whenever *y* = (*y*_*k*_) ∈ *ℓ*_*p*_. This means that *a* = (*a*_*k*_)∈[*ℓ*_*p*_^*λ*^(*B*)]^*β*^ if and only if *D* ∈ (*ℓ*_*p*_ : *c*), where 1 ⩽ *p* ⩽ *∞*. Therefore, we derive from (51) and (52) that

(65) $$\begin{array}{c} \sum_{j=k+1}^{\infty }{\left(\frac{-s}{r}\right)}^{k-j}a_{j}\;\text{exists}\;\text{for}\;\text{each}\;k\in \mathbb{N},\\ \underset{n\in \mathbb{N}}{\sup}\sum_{k}^{n-1}{\left|{\tilde{a}}_{k}\left(n\right)\right|}^{q}<\infty ,\\ \underset{k\in \mathbb{N}}{\sup}\left|\frac{\lambda_{k}}{r\left(\lambda_{k}-\lambda_{k-1}\right)}a_{k}\right|<\infty , \end{array}$$

which shows that [*ℓ*_*p*_^*λ*^(*B*)]^*β*^ = *e*_*q*_^*λ*^∩*d*_1_^*λ*^∩*d*_4_^*λ*^ for 1 < *p* < *∞*. Since beta-dual of the space *ℓ*_*p*_^*λ*^(*B*) for the cases *p* = 1 and *p* = *∞* can be similarly computed, we omit the details. This completes the proof.

**Theorem 16**

Let 1 < *p* ⩽ *∞*. Then, ${\left[\ell_{p}^{\lambda }(B)\right]}^{\gamma }=\{\begin{array}{cc} d_{2}^{\lambda }\;\cap \;d_{4}^{\lambda }, & p\;=\;1,\\ e_{1}^{\lambda }\;\cap \;d_{4}^{\lambda }, & p\;>\;1. \end{array}$

**Proof**

This may be obtained in the similar way used in the proof of Theorem 15 with Lemma 13 instead of Lemma 12. So, we omit the details.

## 6. Certain Matrix Mapping Related to the Spaces *ℓ*_*p*_^*λ*^(*B*) and *ℓ*_*∞*_^*λ*^(*B*)

In this section, we characterize the matrix classes (*ℓ*_*p*_^*λ*^(*B*) : *ℓ*_*∞*_), (*ℓ*_*p*_^*λ*^(*B*) : *c*_0_), (*ℓ*_*p*_^*λ*^(*B*) : *c*), (*ℓ*_*p*_^*λ*^(*B*) : *ℓ*_1_), (*ℓ*_1_^*λ*^(*B*) : *ℓ*_*p*_), (*ℓ*_*∞*_^*λ*^(*B*) : *ℓ*_*p*_), where 1 ⩽ *p* ⩽ *∞*. Also, by means of a given basic lemma, we derive the characterizations of certain other classes. Since the characterization of matrix mapping on the space *ℓ*_*p*_^*λ*^(*B*) can be proved in a similar way, we omit the proof for the cases *p* = 1 and *p* = *∞* and consider only the case 1 < *p* < *∞* in the proofs of theorems given in this section.

For an infinite matrix *A* = (*a*_*nk*_), we write for brevity that

(66) a~nk(m) =λk{ankr(λk−λk−1)+[1r(λk−λk−1)+1s(λk+1−λk)]     ×∑j=k+1m(−sr)k−janj} if  k<m,a~nk =λk{ankr(λk−λk−1)+[1r(λk−λk−1)+1s(λk+1−λk)]     ×∑j=k+1∞(−sr)k−janj},a(n,k,m)=1m+1∑j=0man+j,k ∀n,k,m∈ℕ.

Now, we may begin with quoting the following lemmas (see [22]) which are needed for proving our main results.

**Lemma 17**

Let *A* = (*a*_*nk*_) be an infinite matrix. Then, the following statements hold.

(i) *A* ∈ (*ℓ*_1_ : *c*_0_) if and only if sup⁡_*n*,*k*∈*ℕ*_ | *a*_*nk*_ | <*∞* and
  (67) $$\begin{array}{c} \underset{n\to \infty }{\lim}a_{nk}=0\;for\;\;each\;\;fixed\;\;k\in \mathbb{N}. \end{array}$$
(ii) Let 1 < *p* < *∞*. Then, *A* ∈ (*ℓ*_*p*_ : *c*_0_) if and only if (67) holds and sup⁡_*k*∈*ℕ*_∑_*n*_ | *a*_*nk*_|^*q*^ < *∞*.
(iii) *A* ∈ (*ℓ*_*∞*_ : *c*_0_) if and only if (67) holds and ∑_*k*_ | *a*_*nk*_| is uniformly convergent.

**Lemma 18**

Let 1 ⩽ *p* < *∞*. Then, *A* = (*a*_*nk*_)∈(*ℓ*_1_ : *ℓ*_*p*_) if and only if sup⁡_*n*∈*ℕ*_∑_*k*_ | *a*_*nk*_|^*q*^ < *∞*.

**Lemma 19**

Let 1 < *p* < *∞*. Then, *A* = (*a*_*nk*_)∈(*ℓ*_*∞*_ : *ℓ*_*p*_) if and only if sup⁡_*K*∈*ℱ*_∑_*n*_|∑_*k*∈*K*_ *a*_*nk*_|^*p*^ < *∞*.

**Theorem 20**

Let *A* = (*a*_*nk*_) be an infinite matrix. Then, the following statements hold.

(i) Let 1 < *p* < *∞*. Then, *A* ∈ (*ℓ*_*p*_^*λ*^(*B*) : *ℓ*_*∞*_) if and only if
  (68) $$\begin{array}{c} \sum_{j=k+1}^{\infty }{\left(\frac{-s}{r}\right)}^{k-j}a_{j}\;exists\;\;for\;\;each\;\;fixed\;\;k\in \mathbb{N}, \end{array}$$
  (69) $$\begin{array}{c} \left\{\frac{\lambda_{k}}{r\left(\lambda_{k}-\lambda_{k-1}\right)}a_{nk}\right\}\in \ell_{\infty }\;for\;\;every\;\;n\in \mathbb{N}, \end{array}$$
  (70) $$\begin{array}{c} \underset{k\in \mathbb{N}}{\sup}\sum_{n}{\left|{\tilde{a}}_{nk}\right|}^{q}<\infty . \end{array}$$
  (71) $$\begin{array}{c} {\left(a_{nk}\right)}_{k\in \mathbb{N}}\in e_{q}^{\lambda }\cap d_{1}^{\lambda }\cap d_{4}^{\lambda }\;for\;\;each\;n\in \mathbb{N}. \end{array}$$
(ii) *A* ∈ (*ℓ*_1_^*λ*^(*B*) : *ℓ*_*∞*_) if and only if (68) and (69) hold and
  (72) $$\begin{array}{c} \underset{n,k\in \mathbb{N}}{\sup}\left|{\tilde{a}}_{nk}\right|<\infty . \end{array}$$
(iii) *A* ∈ (*ℓ*_*∞*_^*λ*^(*B*) : *ℓ*_*∞*_) if and only if (68) and (69) hold and
  (73) $$\begin{array}{c} \underset{k\in \mathbb{N}}{\sup}\sum_{n}\left|{\tilde{a}}_{nk}\right|<\infty , \end{array}$$
  (74) $$\begin{array}{c} \underset{m\to \infty }{\lim}\sum_{n}\left|{\tilde{a}}_{nk}\left(m\right)\right|=\sum_{n}\left|{\tilde{a}}_{nk}\right|. \end{array}$$

**Proof**

(i) Assume that the conditions (68)–(71) hold and take any *x* ∈ *ℓ*_*p*_^*λ*^(*B*), where 1 < *p* < *∞*. Then, we have by Theorem 16 that (*a*_*nk*_)_*k*∈*ℕ*_ ∈ [*ℓ*_*p*_^*λ*^(*B*)]^*β*^ for all *n* ∈ *ℕ* and this implies the existence of *Ax*. Also, it is clear that the associated sequence *y* = (*y*_*k*_) is in the space *ℓ*_*p*_ ⊂ *c*_0_.

Let us now consider the following equality derived by using relation (12) from *m*th partial sum of the series ∑_*k*_ *a*_*nk*_ *x*_*k*_ as follows:

(75) ∑k=0mankxk=∑k=0m−1a~nk(m)yk+λmr(λm−λm−1)anmym∀n,m∈ℕ.

Therefore, by using (68)–(70), we obtain from (75) as *m* → *∞* that

(76) $$\begin{array}{c} \sum_{k}a_{nk}x_{k}=\sum_{k}{\tilde{a}}_{nk}y_{k}\;\forall n\in \mathbb{N}. \end{array}$$

Furthermore, since the matrix $\tilde{A}=({\tilde{a}}_{nk})$ is in the class (*ℓ*_*p*_ : *ℓ*_*∞*_) by Lemma 13, we have $\tilde{A}y\in \ell_{\infty }$. Now, by passing to supremum over *n* in (76), we derive by applying Hölder's inequality that

(77) ||Ax||∞sup⁡n∈ℕ|∑kankxk|⩽sup⁡n∈ℕ(∑k|a~nk|q)1/q(∑k|yk|p)1/p<∞,

which shows that *Ax* ∈ *ℓ*_*∞*_ and hence *A* ∈ (*ℓ*_*p*_^*λ*^(*B*) : *ℓ*_*∞*_).

Conversely, assume that *A* = (*a*_*nk*_)∈(*ℓ*_*p*_^*λ*^(*B*) : *ℓ*_*∞*_), where 1 < *p* < *∞*. Then, since (*a*_*nk*_)_*k*∈*ℕ*_ ∈ [*ℓ*_*p*_^*λ*^(*B*)]^*β*^ for all *n* ∈ *ℕ* by the hypothesis, the necessity of (71) is obvious. Since (*a*_*nk*_)_*k*∈*ℕ*_ ∈ [*ℓ*_*p*_^*λ*^(*B*)]^*β*^, (76) holds for all sequences *x* ∈ *ℓ*_*p*_^*λ*^(*B*) and *y* ∈ *ℓ*_*p*_ which are connected by relation (12). Let us now consider the continuous linear functionals *f*_*n*_ on *ℓ*_*p*_^*λ*^(*B*) by

(78) $$\begin{array}{c} f_{n}\left(x\right)=\sum_{k}a_{nk}x_{k}\;\forall n\in \mathbb{N}. \end{array}$$

Then, since *ℓ*_*p*_^*λ*^(*B*) and *ℓ*_*p*_ are norm isomorphic, it should follow with (76) that

(79) $$\begin{array}{c} ||f_{n}||={||{\tilde{A}}_{n}||}_{q}={\left(\sum_{k}{\left|{\tilde{a}}_{nk}\right|}^{q}\right)}^{1/q}, \end{array}$$

for all *n* ∈ *ℕ*. This shows that the functionals defined by the rows of *A* on *ℓ*_*p*_^*λ*^(*B*) are pointwise bounded. Thus, we deduce by Banach-Steinhaus theorem that these functionals are uniformly bounded, which yields that there exists a constant *K* > 0 such that ||*f*_*n*_|| ⩽ *K* for all *n* ∈ *ℕ*. This shows the necessity of the condition (70) which completes the proof of part (i).

**Theorem 21**

Let *A* = (*a*_*nk*_) be an infinite matrix. Then, the following statements hold.

(i) *A* ∈ (*ℓ*_1_^*λ*^(*B*) : *c*) if and only if (68) and (69) hold and
  (80) $$\begin{array}{c} \underset{n\to \infty }{\lim}{\tilde{a}}_{nk}=\alpha_{k}\;for\;\;each\;\;k\in \mathbb{N}. \end{array}$$
(ii) Let 1 < *p* < *∞*. Then, *A* ∈ (*ℓ*_*p*_^*λ*^(*B*) : *c*) if and only if (68)–(71) hold and (80) also holds.
(iii) *A* ∈ (*ℓ*_*∞*_^*λ*^(*B*) : *c*) if and only if (68), (69), and (74) hold, and
  (81) $$\begin{array}{c} \underset{n\to \infty }{\lim}\sum_{k}\left|{\tilde{a}}_{nk}-\alpha_{k}\right|=0. \end{array}$$

**Proof**

We consider only part (ii). Assume that *A* satisfies the conditions (68)–(71) and (80), and *x* ∈ *ℓ*_*p*_^*λ*^(*B*), where 1 < *p* < *∞*. Then, *Ax* exists and by using (80), we have for every *k* ∈ *ℕ* that $|{\tilde{a}}_{nk}{|}^{q}\to |\alpha_{k}{|}^{q}$ as *n* → *∞* which leads us with (70) to the following inequality:

(82) $$\begin{array}{c} \sum_{j=0}^{k}{\left|\alpha_{j}\right|}^{q}⩽\sum_{j=0}^{k}{\left|{\tilde{a}}_{nj}\right|}^{q}=M<\infty , \end{array}$$

which holds for every *k* ∈ *ℕ*. This shows that (*α*_*k*_) ∈ *ℓ*_*q*_. Since *x* ∈ *ℓ*_*p*_^*λ*^(*B*), we have *y* ∈ *ℓ*_*p*_. Therefore, we derive by applying Hölder's inequality that (*α*_*k*_ *y*_*k*_) ∈ *ℓ*_1_ for each *y* ∈ *ℓ*_*p*_.

Now, for any given *ϵ* > 0, choose a fixed *k*_0_ ∈ *ℕ* such that

(83) $$\begin{array}{c} {\left(\sum_{k=k_{0}+1}^{k}{\left|y_{k}\right|}^{p}\right)}^{1/p}⩽\frac{\epsilon }{4M^{1/q}}. \end{array}$$

Then, it follows (80) that there is *m*_0_ ∈ *ℕ* such that

(84) $$\begin{array}{c} \left|\sum_{k=0}^{k_{0}}\left({\tilde{a}}_{nk}-\alpha_{k}\right)y_{k}\right|⩽\frac{\epsilon }{2}, \end{array}$$

for every *m*⩾*m*_0_. Thus, by using (76), we get that

(85) |∑kankxk−∑kαkyk| =|∑k(a~nk−αk)yk| ⩽|∑k=0k0(a~nk−αk)yk|+|∑k=k0+1∞(a~nk−αk)yk|  <ϵ2+[∑k=k0∞(|a~nk|+|αk|)q]1/q[∑k=k0∞|yk|p]1/p  <ϵ2+ϵ4M1/q[(∑k=k0∞(|a~nk|)q)1/q+(∑k=k0∞|αk|p)1/p]  <ϵ2+ϵ4M1/q2M1/q=ϵ,

for all sufficiently large *m*⩾*m*_0_. Hence, (*Ax*)_*n*_ → ∑_*k*_ *α*_*k*_ *y*_*k*_ as *n* → *∞* which means that *Ax* ∈ *c*; that is, *A* = (*a*_*nk*_)∈(*ℓ*_*p*_^*λ*^(*B*) : *c*).

Conversely, suppose that *A* ∈ (*ℓ*_*p*_^*λ*^(*B*) : *c*), where 1 < *p* < *∞*. Then, since *c* ⊂ *ℓ*_*∞*_, *A* ∈ (*ℓ*_*p*_^*λ*^(*B*) : *ℓ*_*∞*_). Thus, the necessity of (68)–(71) is immediately obtained from Theorem 20 which together imply that (76) holds for all sequences *x* ∈ *ℓ*_*p*_^*λ*^(*B*). Since *Ax* ∈ *c* by our assumption, we derive by (76) that $\tilde{A}y\in c$ which means that $\tilde{A}=({\tilde{a}}_{nk})\in (\ell_{p}:c)$. Thus the necessity of (80) is immediate by (51) of Lemma 12. This completes the proof of part (ii).

Now, we can mention the sequence space *f* of almost convergent sequences. The shift operator *P* is defined on *ω* by (*Px*)_*n*_ = *x*_*n*+1_ for all *n* ∈ *ℕ*. A Banach limit *L* is defined on *ℓ*_*∞*_ such that

- *L*(*x*)⩾0 for *x* = (*x*_*k*_), where *x*_*k*_⩾0 for all *k* ∈ *ℕ*,
- *L*(*Px*) = *L*(*x*),
- *L*(*e*) = 1, where *e* = (1,1, 1, etc.).

A sequence *x* = (*x*_*k*_) ∈ *ℓ*_*∞*_ is said to be almost convergent to the generalized limit *α* if all Banach limits of *x* is *α* [23], and denoted by *f* − lim⁡*x*_*k*_ = *α*. Let *P* ^*j*^ be the composition of *P* with itself for *j* times and define *t*_*mn*_(*x*) for a sequence *x* = (*x*_*k*_) by

(86) $$\begin{array}{c} t_{mn}\left(x\right):=\frac{1}{m+1}\sum_{j=0}^{m}{\left(P^{j}x\right)}_{n}\;\forall m,n\in \mathbb{N}. \end{array}$$

Lorentz [23] proved that *f* − lim⁡*x*_*k*_ = *α* if and only if lim⁡_*m*→*∞*_ *t*_*mn*_(*x*) = *α*, uniformly in *n*. It is well-known that a convergent sequence is almost convergent such that its ordinary and generalized limits are equal. By *f* and *fs*, we denote the space of all almost convergent sequences and series, respectively, that is,

(87) f={x=(xk)∈ω:∃α∈ℂ∋lim⁡m→∞∑k=0mxn+km+1=α  uniformly  in  n},fs={x=(xk)∈ω:∃α∈ℂ∋lim⁡m→∞∑k=0m∑j=0n+kxjm+1=α  uniformly  in  n}.

**Theorem 22**

Let *A* = (*a*_*nk*_) be an infinite matrix. Then, the following statements hold.

(i) *A* ∈ (*ℓ*_1_^*λ*^(*B*) : *f*) if and only if (68) and (69) hold, and
  (88) $$\begin{array}{c} f-\underset{n\to \infty }{\lim}{\tilde{a}}_{nk}=\alpha_{k}\;for\;\;every\;\;k\in \mathbb{N}. \end{array}$$
(ii) Let 1 < *p* < *∞*. Then, *A* ∈ (*ℓ*_*p*_^*λ*^(*B*) : *f*) if and only if (68)–(71) and (88) hold.
(iii) *A* ∈ (*ℓ*_*∞*_^*λ*^(*B*) : *f*) if and only if (68) and (69) hold, and
  (89) $$\begin{array}{c} \underset{m\to \infty }{\lim}\sum_{n}\left|\tilde{a}\left(n,k,m\right)-\alpha_{k}\right|=0\;uniformly\;n. \end{array}$$

**Proof**

Theorem 22 can be similarly proved by the same technique used in the proof of Theorem 21.

**Theorem 23**

Let *A* = (*a*_*nk*_) be an infinite matrix. Then, the following statements hold.

(i) *A* = (*a*_*nk*_)∈(*ℓ*_1_^*λ*^(*B*) : *c*_0_) if and only if (68) and (69) hold, and
  (90) $$\begin{array}{c} \underset{n\to \infty }{\lim}{\tilde{a}}_{nk}=0\;for\;\;every\;\;k\in \mathbb{N}. \end{array}$$
(ii) Let 1 < *p* < *∞*. Then, *A* = (*a*_*nk*_)∈(*ℓ*_*p*_^*λ*^(*B*) : *c*_0_) if and only if (68)–(71) and (90) hold.
(iii) *A* = (*a*_*nk*_)∈(*ℓ*_*∞*_^*λ*^(*B*) : *c*_0_) if and only if (68), (69), and (74) hold and
  (91) $$\begin{array}{c} \underset{n\to \infty }{\lim}\sum_{k}\left|{\tilde{a}}_{nk}\right|=0. \end{array}$$

**Proof**

It is natural that Theorem 23 can be proved by the same technique used in the proof of Theorem 21 with Lemma 12 instead of Lemma 17 and so we omit the proof.

**Theorem 24**

Let *A* = (*a*_*nk*_) be an infinite matrix. Then, the following statements hold.

(i) *A* ∈ (*ℓ*_1_^*λ*^(*B*) : *ℓ*_1_) if and only if (68), (69), and (72) hold and
  (92) $$\begin{array}{c} \underset{k\in \mathbb{N}}{\sup}\sum_{n}\left|{\tilde{a}}_{nk}\right|<\infty . \end{array}$$
(ii) Let 1 < *p* < *∞*. Then, *A* ∈ (*ℓ*_*p*_^*λ*^(*B*) : *ℓ*_1_) if and only if (68)–(71) hold and
  (93) $$\begin{array}{c} \underset{F\in ℱ}{\sup}\sum_{k}{\left|\sum_{n\in F}{\tilde{a}}_{nk}\right|}^{q}<\infty . \end{array}$$
(iii) *A* ∈ (*ℓ*_*∞*_^*λ*^(*B*) : *ℓ*_1_) if and only if (68), (69), and (74) hold and
  (94) $$\begin{array}{c} \underset{F\in ℱ}{\sup}\sum_{k}\left|\sum_{n\in F}{\tilde{a}}_{nk}\right|<\infty . \end{array}$$

**Proof**

Since Parts (i) and (iii) can be proved in a similar way, to avoid the repetition of the similar statements, we consider only part (ii).

Suppose that *A* satisfies the conditions (68)–(71), (93) and take any *x* ∈ *ℓ*_*p*_^*λ*^(*B*), where 1 < *p* < *∞*, then *y* ∈ *ℓ*_*p*_. We have by Theorem 16 that (*a*_*nk*_)_*k*∈*ℕ*_ ∈ [*ℓ*_*p*_^*λ*^(*B*)]^*β*^ for all *n* ∈ *ℕ* and this implies that *Ax* exists. Besides, it follows by combining (93) and Lemma 11 that the matrix $\tilde{A}\in (\ell_{p}:\ell_{1})$ and so we have $\tilde{A}y\in \ell_{1}$. Additionally, we derive from (68)–(71) that the relation (76) holds which yields that *Ax* ∈ *ℓ*_1_ and so we have *A* ∈ (*ℓ*_*p*_^*λ*^(*B*) : *ℓ*_1_).

Conversely, assume that *A* ∈ (*ℓ*_*p*_^*λ*^(*B*) : *ℓ*_1_), where 1 < *p* < *∞*. Since *ℓ*_1_ ⊂ *ℓ*_*∞*_, *A* ∈ (*ℓ*_*p*_^*λ*^(*B*) : *ℓ*_*∞*_). Thus, Theorem 20 implies the necessity of (68)–(71) which imply the relation (76). Since *Ax* ∈ *ℓ*_1_ by the hypothesis, we deduce by (76) that $\tilde{A}y\in \ell_{1}$ which means that $\tilde{A}\in (\ell_{p}:\ell_{1})$. Now, the necessity of (93) is immediate by the condition (49) of Lemma 11. This completes the proof of part (ii).

**Theorem 25**

Let 1 ⩽ *p* < *∞*. Then, *A* = (*a*_*nk*_)∈(*ℓ*_1_^*λ*^(*B*) : *ℓ*_*p*_) if and only if (68) and (69) hold, and

(95) $$\begin{array}{c} \underset{k\in \mathbb{N}}{\sup}\sum_{n}{\left|{\tilde{a}}_{nk}\right|}^{p}<\infty . \end{array}$$

**Proof**

Suppose that the conditions (68), (69), and (95) hold and take *x* ∈ *ℓ*_1_^*λ*^(*B*). Then, *y* ∈ *ℓ*_1_. We have by Theorem 16 that (*a*_*nk*_)_*k*∈*ℕ*_ ∈ [*ℓ*_1_^*λ*^(*B*)]^*β*^ for each *n* ∈ *ℕ* and this implies that *Ax* exists. Furthermore, by (95), one can obtain that

(96) $$\begin{array}{c} \underset{k\in \mathbb{N}}{\sup}\left|{\tilde{a}}_{nk}\right|⩽\underset{k\in \mathbb{N}}{\sup}{\left(\sum_{n}|{\tilde{a}}_{nk}{|}^{p}\right)}^{1/p}<\infty \;\text{for}\;\;\text{every}\;n\in \mathbb{N}. \end{array}$$

Hence, the series $\sum_{n}|{\tilde{a}}_{nk}|$ absolutely converges for each fixed *n* ∈ *ℕ*. Therefore; since (68) and (69) hold, if we let to limit in (75) as *m* → *∞*, the relation (76) holds. Thus, by applying Minkowski's inequality and using (76) and (95), we obtain

(97) (∑n|∑kankxk|p)1/p=(∑n|∑ka~nkyk|p)1/p⩽∑kyk(∑n|a~nk|p)1/p<∞,

which means that *Ax* ∈ *ℓ*_*p*_ and so *A* ∈ (*ℓ*_1_^*λ*^(*B*) : *ℓ*_*p*_).

Conversely, assume that *A* ∈ (*ℓ*_1_^*λ*^(*B*) : *ℓ*_*p*_), where 1 ⩽ *p* < *∞*. Since *ℓ*_*p*_ ⊂ *ℓ*_*∞*_, then *A* ∈ (*ℓ*_1_^*λ*^(*B*) : *ℓ*_*∞*_). Thus, Theorem 20 implies that the necessity of (68) and (69) is clear by the relation (76). Since *Ax* ∈ *ℓ*_*p*_ by our assumption, we deduce by (76) that $\tilde{A}y\in \ell_{p}$ which means that $\tilde{A}\in (\ell_{1}:\ell_{p})$. Now, the necessity of (95) is immediate by Lemma 18. This completes the proof.

**Theorem 26**

Let 1 < *p* < *∞*. Then, *A* = (*a*_*nk*_)∈(*ℓ*_*∞*_^*λ*^(*B*) : *ℓ*_*p*_) if and only if (68) and (69) hold and

(98) $$\begin{array}{c} \sum_{k}\left|{\tilde{a}}_{nk}\right|\;converges\;\;for\;\;every\;\;n\in \mathbb{N},\\ \underset{K\in ℱ}{\sup}\sum_{n}{\left|\sum_{k\in K}{\tilde{a}}_{nk}\right|}^{p}<\infty . \end{array}$$

**Proof**

Theorem 26 can be proved by the same technique used in the proof of Theorem 25 with Lemma 19 instead of Lemma 18 and so we omit the details.

**Lemma 27 (see [8, Lemma 5.3])**

Let *λ* and *μ* be any two sequence spaces, let *A* be an infinite matrix and *B* a triangle matrix. Then, *A* ∈ (*λ* : *μ*_*B*_) if and only if *BA* ∈ (*λ* : *μ*).

It is trivial that Lemma 27 has several consequences. Indeed, combining Lemma 27 with Theorems 20–26, one can derive the following results.

**Corollary 28**

Let *A* = (*a*_*nk*_) be an infinite matrix and *u* = (*u*_*n*_) and *v* = (*v*_*n*_) be sequences of non-zero numbers, and define the matrix *C* = (*c*_*nk*_) by *c*_*nk*_ = *u*_*n*_∑_*j*=0_^*n*^ *v*_*j*_ *a*_*jk*_ for all *n*, *k* ∈ *ℕ*. Then, the necessary and sufficient conditions in order *A* belongs to any of the classes (*ℓ*_*p*_^*λ*^(*B*) : *ℓ*_*∞*_(*u*, *v*)), (*ℓ*_*p*_^*λ*^(*B*) : *ℓ*_*p*_(*u*, *v*)), and (*ℓ*_*p*_^*λ*^(*B*) : *c*(*u*, *v*)) are obtained from respective ones in Theorems 20–26 by replacing the entries of the matrix *A* by those of the matrix *C*. The spaces *ℓ*_*∞*_(*u*, *v*), *ℓ*_*p*_(*u*, *v*), and *c*(*u*, *v*) are defined in [9] as the spaces of all sequences whose generalized weighted means are in the spaces *ℓ*_*∞*_, *c*, and *ℓ*_*p*_. Since the spaces *ℓ*_*∞*_(*u*, *v*), *c*(*u*, *v*), and *ℓ*_*p*_(*u*, *v*) can be reduce in the cases *v*_*k*_ = *r*_*k*_, *u*_*n*_ = 1/*R*_*n*_ and *v*_*k*_ = 1, *u*_*n*_ = 1/*n* to the Riesz sequence spaces *r*_*∞*_^*t*^, *r*_*c*_^*t*^, and *r*_*p*_^*t*^ and to the Cesàro sequence spaces *X*_*∞*_, $\tilde{c}$, and *X*_*p*_, respectively, Corollary 28 also includes the characterizations of classes (*ℓ*_*p*_^*λ*^(*B*) : *r*_*∞*_^*t*^), (*ℓ*_*p*_^*λ*^(*B*) : *r*_*p*_^*t*^), (*ℓ*_*p*_^*λ*^(*B*) : *r*_*c*_^*t*^) and (*ℓ*_*p*_^*λ*^(*B*) : *X*_*∞*_), (*ℓ*_*p*_^*λ*^(*B*) : *X*_*p*_) and $\left(\ell_{p}^{\lambda }(B):\tilde{c}\right)$, where 1 ⩽ *p* ⩽ *∞*.

**Corollary 29**

Let *A* = (*a*_*nk*_) be an infinite matrix and define the matrix *C* = (*c*_*nk*_) by

(99) $$\begin{array}{c} c_{nk}=\sum_{j=0}^{n}\left(\begin{array}{c} n\\ j \end{array}\right){\left(1-r\right)}^{n-j}r^{-j}a_{jk}\;\forall n,k\in \mathbb{N}. \end{array}$$

Then, the necessary and sufficient conditions in order *A* which belongs to any of the classes (*ℓ*_*p*_^*λ*^(*B*) : *e*_*∞*_^*r*^), (*ℓ*_*p*_^*λ*^(*B*) : *e*_0_^*r*^), (*ℓ*_*p*_^*λ*^(*B*) : *e*_*p*_^*r*^), and (*ℓ*_*p*_^*λ*^(*B*) : *e*_*c*_^*r*^) are obtained from respective ones in Theorems 20–26 by replacing the entries of the matrix *A* by those of the matrix *C*; where *e*_*∞*_^*r*^, *e*_*p*_^*r*^ and *e*_*c*_^*r*^, and *e*_0_^*r*^ denote the Euler spaces of all sequences whose *E* ^*r*^-transforms are in the spaces *ℓ*_*∞*_, *ℓ*_*p*_ and *c*, and *c*_0_ which were introduced in [6, 12], where 1 ⩽ *p* < *∞*.

## 7. Some Geometric Properties of the Space *ℓ*_*p*_^*λ*^(*B*)

In the present section, we investigate some geometric properties of the space *ℓ*_*p*_^*λ*^(*B*). First, we define some geometric properties of the spaces. Let (*X*, ||·||) be a normed space and let *S*(*x*) and *B*(*x*) be the unit sphere and unit ball of *X*, respectively. Consider *Clarkson's modulus of convexity* (see [24, 25]) defined by

(100) $$\begin{array}{c} \delta_{X}\left(\varepsilon \right)=\inf\left\{1-\frac{||x-y||}{2};\;x,y\in S\left(x\right),\;||x-y||=\varepsilon \right\}, \end{array}$$

where 0 ⩽ *ε* ⩽ 2. The inequality *δ*_*X*_(*ε*) > 0 for all *ε* ∈ [0,2] characterizes the uniformly convex spaces. In [26], *Gurarii's modulus of convexity* is defined by

(101) βX(ε)=inf⁡{1−inf⁡α∈[0,1]||αx+(1−α)y||;  x,y∈S(x),  ||x−y||=ε},

where 0 ⩽ *ε* ⩽ 2. It is easily shown that *δ*_*X*_(*ε*) ⩽ *β*_*X*_(*ε*) ⩽ 2*δ*_*X*_(*ε*) for any 0 ⩽ *ε* ⩽ 2. Further, if 0 < *β*_*X*_(*ε*) < 1, then *X* is uniformly convex, and if *β*_*X*_(*ε*) < 1, then *X* is strictly convex.

A Banach space *X* is said to have the *Banach-Saks property* if every bounded sequence (*x*_*n*_) in *X* admits a sequence (*z*_*n*_) such that the sequence {*t*_*k*_(*z*)} is convergent in the norm in *X* [27], where

(102) $$\begin{array}{c} t_{k}\left(z\right)=\frac{1}{k+1}\left(z_{0}+z_{1}+\cdots +z_{k}\right)\;\forall k\in \mathbb{N}. \end{array}$$

A Banach space *X* is said to have the *weak Banach-Saks property* whenever given any weakly null sequence (*x*_*n*_) in *X* and there exists a subsequence (*z*_*n*_) of (*x*_*n*_) such that the sequence {*t*_*k*_(*z*)} is strongly convergent to zero.

In [28], García-Falset introduced the following coefficient:

(103) $$\begin{array}{c} R\left(X\right)=\sup\left\{\underset{n\to \infty }{\lim\inf}||x_{n}+x||;\left(x_{n}\right)\subset B\left(x\right),x_{n}\overset{w}{\to }0\right\}. \end{array}$$

**Remark 30 (see [29])**

A Banach space *X* with *R*(*X*) < 2* has a weak fixed point property. *

**Theorem 31**

The space *ℓ*_*p*_^*λ*^(*B*) has Banach-Saks type *p*.

**Proof**

Let (*ε*_*n*_) be a sequence of positive numbers for which ∑_*n*=1_^*∞*^ *ε*_*n*_ ⩽ 1/2. Let (*x*_*n*_) be a weakly null sequence in *B*(*ℓ*_*p*_^*λ*^(*B*)). Set *u*_0_ = *x*_0_ and *u*_1_ = *x*_*n*_1__ = *x*_1_. Then, there exists *t*_1_ ∈ *ℕ* such that

(104) $$\begin{array}{c} {||\sum_{i=t_{1}+1}^{\infty }u_{1}\left(i\right)e^{\left(i\right)}||}_{\ell_{p}^{\lambda }(B)}<\varepsilon_{1}. \end{array}$$

The assumption “(*x*_*n*_) is a weakly null sequence” implies that *x*_*n*_ → 0 with respect to the coordinatewise, there exists *n*_2_ ∈ *ℕ* such that

(105) $$\begin{array}{c} {||\sum_{i=0}^{t_{1}}x_{n}\left(i\right)e^{\left(i\right)}||}_{\ell_{p}^{\lambda }(B)}<\varepsilon_{1}, \end{array}$$

where *n*⩾*n*_2_. Set *u*_2_ = *x*_*n*_2__. Then, there exists *t*_2_ > *t*_1_ such that

(106) $$\begin{array}{c} {||\sum_{i=t_{2}+1}^{\infty }u_{2}\left(i\right)e^{\left(i\right)}||}_{\ell_{p}^{\lambda }(B)}<\varepsilon_{2}. \end{array}$$

By using the fact that *x*_*n*_ → 0 with respect to the coordinatewise, there exists *n*_3_ > *n*_2_ such that

(107) $$\begin{array}{c} {||\sum_{i=0}^{t_{2}}x_{n}\left(i\right)e^{\left(i\right)}||}_{\ell_{p}^{\lambda }(B)}<\varepsilon_{2}, \end{array}$$

where *n*⩾*n*_3_. If we continue this process, we can find two increasing sequences (*t*_*i*_) and (*n*_*i*_) of natural numbers such that

(108) $$\begin{array}{c} {||\sum_{i=0}^{t_{j}}x_{n}\left(i\right)e^{\left(i\right)}||}_{\ell_{p}^{\lambda }(B)}<\varepsilon_{j}, \end{array}$$

for each *n*⩾*n*_*j*+1_ and

(109) $$\begin{array}{c} {||\sum_{i=t_{j}+1}^{\infty }u_{j}\left(i\right)e^{\left(i\right)}||}_{\ell_{p}^{\lambda }(B)}<\varepsilon_{j}, \end{array}$$

where *u*_*j*_ = *x*_*n*_*j*__. Hence,

(110) ||∑j=0nuj||ℓpλ(B)=||∑j=0n(∑i=0tj−1uj(i)e(i)+∑i=tj−1tjuj(i)e(i)+∑i=tj+1∞uj(i)e(i))||ℓpλ(B)⩽||∑j=0n∑i=0tj−1uj(i)e(i)||ℓpλ(B)+||∑j=0n ∑i=tj−1tjuj(i)e(i)||ℓpλ(B)+||∑j=0n ∑i=tj+1∞uj(i)e(i)||ℓpλ(B)⩽||∑j=0n(∑i=tj−1+1tjuj(i)e(i))||ℓpλ(B)+2∑j=0nεj.

On the other hand, one can see that ||*x*||_*ℓ*_*p*_^*λ*^(*B*)_ < 1. Thus, ||*x*||_*ℓ*_*p*_^*λ*^(*B*)_^*p*^ < 1, and we have

(111) ||∑j=0n ∑i=tj−1+1tjuj(i)e(i)||ℓpλ(B)p =∑j=0n ‍∑i=tj−1tj|∑k=0i−1a~ikxj(k)+λir(λi−λi−1)xi(k)|p  +∑j=0n‍ ∑i=0∞|∑k=0ia~ikxj(k)+λir(λi−λi−1)xi(k)|p ⩽(n+1).

Therefore, we obtain

(112) $$\begin{array}{c} {||\sum_{j=0}^{n}\;‍\sum_{i=t_{j-1}+1}^{t_{j}}u_{j}\left(i\right)e^{\left(i\right)}||}_{\ell_{p}^{\lambda }(B)}{⩽\left(n+1\right)}^{1/p}. \end{array}$$

By using that fact that 1 ⩽ (*n* + 1)^1/*p*^ for all *n* ∈ *ℕ* and 1 ⩽ *p* < *∞*, we have

(113) $$\begin{array}{c} {||\sum_{j=0}^{n}u_{j}||}_{\ell_{p}^{\lambda }(B)}⩽{\left(n+1\right)}^{1/p}+1⩽2{\left(n+1\right)}^{1/p}. \end{array}$$

Therefore, the space *ℓ*_*p*_^*λ*^(*B*) has Banach-Saks type *p*.

**Remark 32**

Note that *R*(*ℓ*_*p*_^*λ*^(*B*)) = *R*(*ℓ*_*p*_) = 2^1/*p*^, since *ℓ*_*p*_^*λ*^(*B*) is linearly isomorphic to *ℓ*_*p*_.

Thus, by Remarks 30 and 32, we have the following.

**Corollary 33**

Let 1 < *p* < *∞*. Then, the sequence space *ℓ*_*p*_^*λ*^(*B*) has the weak fixed point property.

**Theorem 34**

Gurarii's modulus of convexity for the normed space *ℓ*_*p*_^*λ*^(*B*) is

(114) $$\begin{array}{c} \beta_{\ell_{p}^{\lambda }(B)}\left(\varepsilon \right)⩽1-{\left[1-{\left(\frac{\varepsilon }{2}\right)}^{p}\right]}^{1/p}, \end{array}$$

where 0 ⩽ *ε* ⩽ 2.

**Proof**

Let *x* ∈ *ℓ*_*p*_^*λ*^(*B*). Then, we have

(115) $$\begin{array}{c} {||x||}_{\ell_{p}^{\lambda }(B)}={||\hat{\Lambda }x||}_{p}={\left[\sum_{n}{\left|{\left(\hat{\Lambda }x\right)}_{n}\right|}^{p}\right]}^{1/p}. \end{array}$$

Let 0 ⩽ *ε* ⩽ 2 and consider the following sequences:

(116) $$\begin{array}{c} z=\left(z_{n}\right)=\left\{{\hat{\Lambda }}^{-1}\left[{\left[1-{\left(\frac{\varepsilon }{2}\right)}^{p}\right]}^{1/p}\right],{\hat{\Lambda }}^{-1}\left(\frac{\varepsilon }{2}\right),0,0,\ldots \right\},\\ t=\left(t_{n}\right)=\left\{{\hat{\Lambda }}^{-1}\left[{\left[1-{\left(\frac{\varepsilon }{2}\right)}^{p}\right]}^{1/p}\right],{\hat{\Lambda }}^{-1}\left(-\frac{\varepsilon }{2}\right),0,0,\ldots \right\}. \end{array}$$

Since $u_{n}={\left(\hat{\Lambda }z\right)}_{n}$ and $v_{n}={\left(\hat{\Lambda }t\right)}_{n}$, one can see that

(117) $$\begin{array}{c} u=\left(u_{n}\right)=\left\{{\left[1-{\left(\frac{\varepsilon }{2}\right)}^{p}\right]}^{1/p},\frac{\varepsilon }{2},0,0,\ldots \right\},\\ v=\left(v_{n}\right)=\left\{{\left[1-{\left(\frac{\varepsilon }{2}\right)}^{p}\right]}^{1/p},-\frac{\varepsilon }{2},0,0,\ldots \right\}. \end{array}$$

By using the sequences *z* = (*z*_*n*_) and *t* = (*t*_*n*_), we obtain the following equalities:

(118) ||z||ℓpλ(B)p=||Λ^z||p=|[(1−ε2)p]p|1/p+|ε2|p=1−(ε2)p+(ε2)p=1,||t||ℓpλ(B)p=||Λ^t||p=|[(1−ε2)p]p|1/p+|−ε2|p=1−(ε2)p+(ε2)p=1,||z−t||ℓpλ(B)p=||Λ^z−Λ^t||p={|[1−(ε2)p]1/p−[1−(ε2)p]1/p|p +|ε2−(−ε2)|p}1/p=ε.

For 0 ⩽ *α* ⩽ 1

(119) inf⁡α∈[0,1]||αz+(1−α)t||ℓpλ(B) =inf⁡α∈[0,1]||αΛ^z+(1−α)Λ^t||p =inf⁡α∈[0,1]{|α[1−(ε2)p]1/p+(1−α)[1−(ε2)p]1/p|p+|αε2+(1−α)(−ε2)|p}1/p =inf⁡α∈[0,1][1−(ε2)p+(2α−1)p(ε2)p]1/p =[1−(ε2)p]1/p.

Therefore, for 1 ⩽ *p* < *∞*, we have

(120) $$\begin{array}{c} \beta_{\ell_{p}^{\lambda }(B)}\left(\varepsilon \right)⩽1-{\left[1-{\left(\frac{\varepsilon }{2}\right)}^{p}\right]}^{1/p}. \end{array}$$

This step concludes the proof.

**Corollary 35**

The following statements hold.

- For *ε* > 2, *β*_*ℓ*_*p*_^*λ*^(*B*)_(*ε*) = 1. Thus, *ℓ*_*p*_^*λ*^(*B*) is strictly convex.
- For 0 < *ε* ⩽ 2, *β*_*ℓ*_*p*_^*λ*^(*B*)_(*ε*) ⩽ 1. Thus, *ℓ*_*p*_^*λ*^(*B*) is uniformly convex.

**Corollary 36**

For *α* = 1/2, *β*_*ℓ*_*p*_^*λ*^(*B*)_(*ε*) = *δ*_*ℓ*_*p*_^*λ*^(*B*)_(*ε*).

## 8. Conclusion

The domain of Euler means *E* ^*r*^ of order *r*, the method *A* ^*r*^, and the generalized difference matrix *B*(*r*, *s*) in the sequence spaces *ℓ*_*p*_ and *ℓ*_*∞*_ investigated by Altay et al. [6], Aydın and Başar [7], and Kirişçi and Başar [30], respectively. Since $\hat{\Lambda }$ is the composition of Λ and *B*(*r*, *s*), our corresponding results are much more general than the results given by Kirişçi and Başar [30]. Additionally, we emphasize on some geometric properties of the new space *ℓ*_*p*_^*λ*^(*B*). It is obvious that the matrix $\hat{\Lambda }$ is not comparable with the matrices *E* ^*r*^, *A* ^*r*^, or *B*(*r*, *s*). So, the present results are new. As a natural continuation of this paper, one can study the domain of the matrix $\hat{\Lambda }$ in Maddox's spaces *ℓ*_*∞*_(*p*), *c*(*p*), *c*_0_(*p*), and *ℓ*(*p*).
